# Prognostic and Clinical Significance of Aspartate Aminotransferase-to-Lymphocyte Ratio Index in Individuals with Liver Cancer: A Meta-Analysis

**DOI:** 10.1155/2022/3533714

**Published:** 2022-02-09

**Authors:** Xiulan Peng, Yali Huang, Min Zhang, Yan Chen, Lihua Zhang, Anbing He, Renfeng Luo

**Affiliations:** ^1^Department of Oncology, The Second Affiliated Hospital of Jianghan University, China; ^2^Department of Neurology, Wuhan Fourth Hospital, Puai Hospital, Tongji Medical College, Huazhong University of Science and Technology, China; ^3^Department of Neurosurgery, Wuhan Fourth Hospital, Puai Hospital, Tongji Medical College, Huazhong University of Science and Technology, China; ^4^Department of Diagnostics, Jianghan University, China

## Abstract

**Objective:**

This study was aimed at exploring the prognostic and clinicopathological roles of aspartate aminotransferase-to-lymphocyte ratio index (ALRI) in patients with hepatocellular carcinoma via a meta-analysis.

**Methods:**

The PubMed, Embase, Cochrane Library, Web of Science, China National Knowledge Infrastructure (CNKI), Wanfang, and VIP databases were comprehensively searched from inception to November 20, 2021. Pooled hazard ratio (HR) and corresponding 95% confidence interval (CI) were used to evaluate the relationship between ALRI and overall survival (OS) as well as progression-free survival (PFS) in patients with hepatocellular carcinoma. Odds ratio (OR) and the corresponding 95% CI were also used to investigate correlations between clinical factors and ALRI in patients with hepatocellular carcinoma.

**Results:**

A total of 3914 patients with hepatocellular carcinoma from eleven retrospective cohorts were included in this meta-analysis. The combined results revealed that patients with hepatocellular carcinoma with elevated ALRI tended to have unfavorable OS (HR 1.53 [95% CI 1.25–1.82]; *P* < 0.001). Pooled HRs revealed that high ALRI was an independent risk factor for inferior PFS in patients with hepatocellular carcinoma (HR 1.36 [95% CI 1.10–1.63]; *P* < 0.001). In addition, high ALRI was strongly associated with male sex (OR 1.32 [95% CI 1.02–1.70]; *P* = 0.035), presence of cirrhosis (OR 1.68 [95% CI 1.01–2.81]; *P* = 0.046), larger tumor size (OR 2.25 [95% CI 1.31–3.88]; *P* < 0.001), presence of portal vein tumor thrombus (OR 2.50 [95% CI 1.52–4.11]; *P* < 0.001), and distant metastasis (OR 1.72 [95% CI 1.05-2.82]; *P* = 0.031).

**Conclusion:**

Elevated ALRI in patients with hepatocellular carcinoma predicted inferior survival outcomes and was strongly associated with some important features of hepatocellular carcinoma.

## 1. Introduction

Hepatocellular carcinoma (HCC) is the most frequent cause of cancer-related death worldwide and ranks fifth in terms of incidence in the United States [1]. Unfortunately, the incidence of HCC continues to increase annually [2]. Recognized risk factors for HCC include hepatitis virus infection, alcohol-related cirrhosis, fatty liver disease, nonalcoholic fatty liver disease, obesity, diabetes, and various dietary exposures [3]. Because of the high rate of hepatitis B virus (HBV) infection among the Chinese population, the HCC causes heavy medical and economic burden in China, and China accounts for approximately 50% of HCC cases worldwide [4]. The prognosis of individuals with HCC is far from satisfactory partially due to the lack of accurate prognostic biomarkers. The primary role of prognostic indexes is to give an estimation of the aggressiveness of HCC on a case-by-case basis. The promising biomarkers related to HCC could be applied appropriately to stratify patients, thus enabling more accurate treatment allocation. Therefore, there is a highly urgent medical demand to identify reliable prognostic biomarkers, especially for HCC, that would be conducive to the design and development of optimal treatment regimens and improve the clinical outcomes of individuals with HCC.

Recent studies have demonstrated that inflammatory response plays an essential role in the progression and metastasis of HCC [5–7]. Therefore, a panel of serum biomarkers based on inflammation parameters from routine bloodwork, such as systemic immune-inflammation index (SII) [8], prognostic nutritional index (PNI) [9], platelet-to-lymphocyte ratio [10], and Integrated Liver Inflammatory Score [11], have been demonstrated to be efficient prognostic biomarkers for patients with cancer. The aspartate aminotransferase- (AST-) to-lymphocyte ratio index (ALRI) is a novel inflammatory index for HCC [12] and is derived from the ratio of AST to lymphocyte count. ALRI has been reported to be related to the survival of patients with HCC [13–23]; however, results have not been consistent across studies. Therefore, we conducted the current meta-analysis to determine the prognostic impact and clinical significance of ALRI in patients with HCC by aggregating all available data.

## 2. Methods

### 2.1. Search Strategy

This meta-analysis was prospectively enrolled in PROSPERO (ID: CRD42021238765, https://www.crd.york.ac.uk/PROSPERO/) and was performed according to the Preferred Reporting Items for Systematic Reviews and Meta-analyses (i.e., PRISMA) statement [24]. The PubMed, Embase, Cochrane Library, Web of Science, China National Knowledge Infrastructure (CNKI), Wanfang, and VIP databases were comprehensively searched from inception to November 20, 2021. Search terms included “liver cancer,” “liver neoplasm,” “hepatocellular carcinoma,” “HCC” AND “aspartate aminotransferase/lymphocyte,” “aspartate aminotransferase-to-lymphocyte ratio index,” “aspartate aminotransferase to lymphocyte ratio,” and “ALRI.” Additionally, the references of relevant studies were manually screened to identify additional potentially eligible studies. The included studies were restricted to those published in English and Chinese.

### 2.2. Inclusion and Exclusion Criteria

Study inclusion criteria were as follows: primary HCC was the only cancer diagnosis; age >18 years; individuals with HCC were classified into two groups based on an ALRI cut-off value; investigation of the association between ALRI and overall survival (OS) or progression-free survival (PFS) among patients with HCC; and reported hazard ratio (HR) and corresponding 95% confidence interval (CI) for ALRI. Reviews, meta-analyses, letters, case reports, clinical studies without full text, or studies not reporting HR and 95% CI for ALRI were excluded.

### 2.3. Data Collection and Quality Evaluation

Two investigators (YH and MZ) independently reviewed the articles retrieved in the literature search and extracted the relevant data. Any disagreements were ultimately resolved by discussion with a third reviewer (RL). The following clinical information was extracted from the retrieved articles by LZ and YC: year of publication; author's surname; study period; study design; staging criteria; age of participants; country of origin, sex; sample size; tumor stage; treatment plan; cut-off value for ALRI; selection of cut-off value; follow-up; and HR and 95% CI for ALRI. The HRs in this meta-analysis were derived from multivariate Cox analysis.

The quality of all included studies was assessed using the Newcastle–Ottawa Scale (NOS) [25]. The NOS rating generally ranges from 0 to 9, and studies with a score >7 were regarded to be of high quality.

### 2.4. Statistical Analysis

In this meta-analysis, all statistical analyses were performed using STATA version 15.0 (STATACorp LLC, College Station, TX, USA). The overall HRs and 95% CIs were calculated to determine the relationship between ALRI and OS or PFS in patients with HCC. Pooled ORs with 95% CIs were also calculated to investigate the correlation between ALRI and common clinical features of HCC. Cochran's *Q* test combined with the *I*^2^ test was used to assess the statistical heterogeneity across the included studies, with significant heterogeneity viewed as *I*^2^ > 50%. A random-effect model was used for pooled data analysis when significant heterogeneity (i.e., *I*^2^ > 50%) was observed; otherwise, a fixed effect model was used. Sensitivity analysis was used to assess the stability of the pooled ORs or HRs by sequentially excluding one study from the analysis. Begg's test together with Egger's test was used to explore for the presence of publication bias.

## 3. Results

### 3.1. Study Characteristics

**Figure 1:**
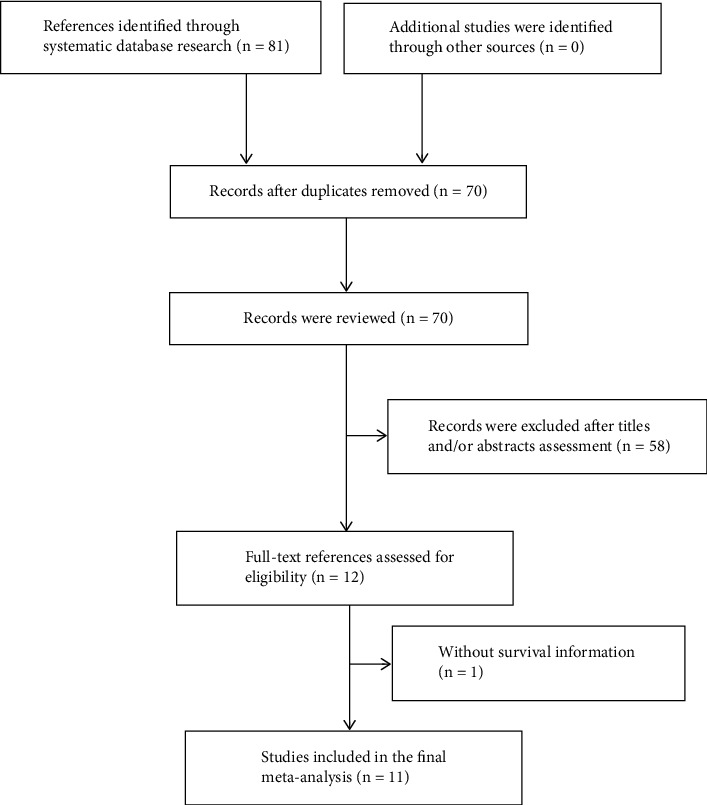
The flow diagram of literature selection.

The primary literature search retrieved 81 articles. After removing duplicate publications and reviewing the abstracts, only eleven clinical studies [13–23] that fulfilled the inclusion criteria were ultimately included in this meta-analysis. The process of the literature selection is presented in Figure 1. The eleven clinical studies included retrospective cohorts and were published between 2015 and 2021. Sample sizes ranged from 78 to 983, with a total of 3914. Interestingly, all eleven studies were performed in China. Three clinical trials were published in the Chinese language [18–20] and the remainder in English. The cut-off value for ALRI ranged from 18.734 to 86, with a mean value of 33.96. All eleven clinical studies reported the association between ALRI and OS, and only seven studies [18–20, 22, 23] demonstrated a correlation between ALRI and PFS. Regarding quality evaluation, only two studies [13, 15] scored 7 and nine studies scored 8; as such, all studies were regarded to be of high quality (Table S1). Detailed clinical information of the nine included studies are summarized in Table 1.

### 3.2. Pooled Analysis of the Association between ALRI and OS

**Figure 2:**
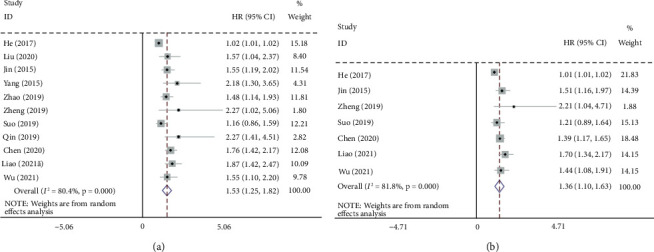
Forest plots of the hazard ration evaluating the association between the ALRI and survival in liver cancer individuals. (a) Overall survival and (b) progression-free survival.

A total of 3914 patients with HCC from eleven retrospective cohorts were included in this meta-analysis. Because of notable study heterogeneity (*I*^2^ = 80.4% and *P* < 0.0001), the random-effect model was selected for the combined meta-analysis. The combined results revealed that patients with HCC with elevated ALRI tended to have unfavorable OS (HR 1.53 [95% CI 1.25–1.82]; *P* < 0.001]) (Figure 2(a)). Owing to the presence of significant heterogeneity in the meta-analysis, subgroup analyses were performed based on a list of common clinical factors. As shown in Table 2, significant correlations between ALRI and inferior OS persisted in the subgroup analyses of treatment, staging criteria, sample size, ALRI cut-off value, cut-off selection, and NOS score.

F: female; M: male; NA: not available; TACE: transcatheter arterial chemoembolization; BCLC: Barcelona clinic hepatocellular carcinoma; AJCC: American Joint Committee on Cancer; NOS: Newcastle–Ottawa Scale; OS: overall survival; PFS: progression-free survival; ROC: receiver-operating characteristics curve. Age were presented as means; follow-up months were presented as median value, while “∗” was presented as means.

### 3.3. Pooled Analysis of the Correlation between ALRI and PFS

Seven studies including 2865 patients with HCC reported information addressing the relationship between ALRI and PFS. Because of the notable heterogeneity (*I*^2^ = 81.8% and *P* < 0.0001), the random-effect model was selected for the combined meta-analysis. The overall results revealed a significant relationship between elevated ALRI and worse PFS among individuals with HCC (HR 1.53 [95% CI 1.25–1.82]; *P* < 0.001]) (Figure 2(b)). As shown in Table 2, remarkable relationships between high ALRI and unfavorable PFS persisted in the subgroup analyses of treatment, staging criteria, sample size, ALRI cut-off value, cut-off selection, and NOS score.

CI: confidence interval; HR: hazard ratio; NA: not available; TACE: transcatheter arterial chemoembolization; BCLC: Barcelona clinic hepatocellular carcinoma; AJCC: American Joint Committee on Cancer; NOS: Newcastle–Ottawa Scale; OS: overall survival; PFS: progression-free survival; ROC: receiver-operating characteristics curve.

### 3.4. Relationship between ALRI and Clinical Features

Based on the clinical characteristics reported in the eleven articles, the clinical significance of ALRI among patients with HCC was further analyzed. The overall OR and corresponding 95% CI were calculated using the STATA software to assess the associations between ALRI and selected clinical characteristics, including sex (male vs. female), age (>60 years vs. ≤60 years), hepatitis B surface antigen (positive vs. negative), alpha-fetoprotein level (>400 vs. ≤400 ng/mL), cirrhosis (yes vs. no), tumor size (≥5 cm vs. <5 cm), tumor number (multiple vs. single), TNM stage (III-IV vs. I-II), portal vein tumor thrombus (yes vs. no), and distant metastasis (yes vs. no). As shown in Table 3, high ALRI was closely associated with male sex (OR 1.32 [95% CI 1.02–1.70]; *P* = 0.035), presence of cirrhosis (OR 1.68 [95% CI 1.01–2.81]; *P* = 0.046), larger tumor size (OR 2.25 [95% CI 1.31–3.88]; *P* < 0.001), presence of portal vein tumor thrombus (OR 2.50 [95% CI 1.52–4.11]; *P* < 0.001), and distant metastasis (OR 1.72 [95% CI 1.05–2.82]; *P* = 0.031).

HBsAg: hepatitis B surface antigen; AFP: alpha-fetoprotein; TNM: tumor-node-metastasis; PVTT: portal vein tumor thrombus; OR: odds ratio; CI: confidence interval.

### 3.5. Sensitivity Analysis

**Figure 3:**
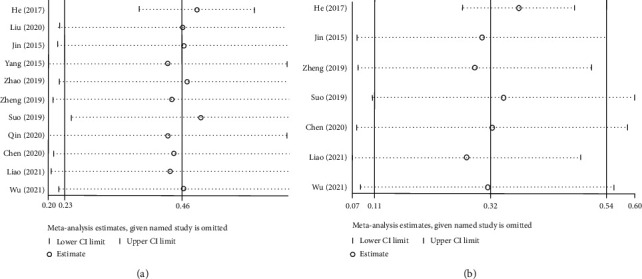
Sensitivity analyses of the impact of ALRI on the survival time among individuals with liver cancer. (a). Overall survival and (b) progression-free survival.

To assess the robustness of the pooled HRs with 95% CI, a sensitivity analysis was performed by sequentially excluding one study at a time from the meta-analysis. The sensitivity analyses indicated that the pooled relationship (i.e., HR) between ALRI and OS was not affected by the removal of any single study (Figure 3(a)). In addition, the results of the sensitivity analyses implied that the overall results of PFS were not significantly altered by the removal of any single study (Figure 3(b)). In other words, results related to OS and PFS in the meta-analysis were robust.

### 3.6. Publication Bias

**Figure 4:**
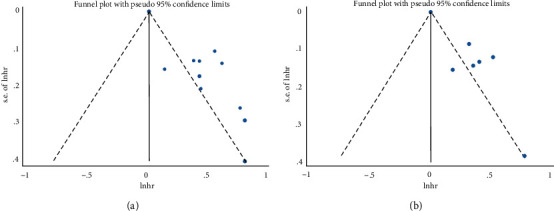
Funnel plots for the evaluation of publication bias. (a) Overall survival and (b) progression-free survival.

Begg's and Egger's tests were used to detect the presence of publication bias. As shown in the funnel plots (Figure 4(a)), no significant publication bias (*P* = 0.533) was observed related to the association between ALRI and OS in the current analysis. However, significant publication bias was noticed based on Egger's test (*P* < 0.05). Moreover, the funnel plot of association between ALRI and PFS showed an asymmetry of the result (Figure 4(b); Begg's test *P* = 0.548), and a significant publication bias was observed in Egger's test (*P* = 0.002).

## 4. Discussion

Recently, an increasing number of serum markers, such as PNI, SII, neutrophil-to-lymphocyte ratio, and monocyte-to-lymphocyte ratio, have been used in the clinical practice owing to their ready availability and cost-effectiveness [26–28]. Among them, ALRI is a novel combined indicator, although its prognostic value and clinical significance in HCC remain uncertain. To our knowledge, the present meta-analysis was the first to determine the overall prognostic impact and clinical significance in HCC by aggregating available data from nine clinical studies.

The results of our meta-analysis have some clinical implications. Based on the available data (*N* = 3914), higher ALRI values were associated with inferior survival—for both OS and PFS—in patients with HCC. Our meta-analysis indicated that patients with HCC with increased ALRI had a higher risk for larger tumor size, presence of portal vein tumor thrombus, and distant metastasis, which would shorten survival time. Because of ready availability and low cost, ALRI could be routinely used to monitor disease progression in individuals with HCC. Our study was helpful for the oncologists in the risk assessment among patients with HCC based on ALRI and also helping them in designing and developing optimal treatment strategies. Individuals with HCC who have higher ALRI before treatment may benefit from radical treatments, such as postoperative adjuvant chemoradiotherapy and/or surgery, than those who have lower ALRI.

Because AST is highly sensitive to damaged liver function, serum AST levels are commonly used to evaluate liver function [29]. Once hepatocytes are damaged/injured, intracellular AST is directly released into the peripheral blood, which leads to increased serum AST levels. Increased AST levels generally imply the activity of HBV in liver cancer patients infected with the virus, and the activity of HBV is a risk factor for decreased survival time in patients with HCC [30]. The close correlation between the pathogenesis of malignant tumor and systemic inflammation is quite common in patients with HCC because most result from chronic hepatitis [31]. Accumulating evidence has revealed that lymphocytes play a central role in the antitumor immune response [32, 33]. CD4+ T helper 1 cells and CD8+ cytotoxic T lymphocytes can kill HCC cells and effectively prevent oncogenesis and progression of HCC [34]. Hence, increased lymphocyte counts signify a relatively favorable prognosis in individuals with HCC. In summary, elevated serum AST levels are strongly associated with the progression of HCC, and a decrease in lymphocyte count reflects damage to antitumor immunity. Therefore, high ALRI suggests worse prognosis in individuals with HCC.

The survival outcomes of HCC mainly depend on the prognostic biomarkers and staging systems, while it is not sufficient to assess the survival of patients with HCC by these criteria alone, as the survival outcome of HCC is not only affected by the tumor biology but also affected by the individuals' liver function. So, ALRI seems to be a promising biomarker for patients with HCC. In addition to the prognostic relevance, ALRI also exhibited clinical relevance with significant clinical features, such as distant metastases, portal vein thrombosis, and tumor size greater than 5 cm.

Our meta-analysis had four obvious limitations. First, all eleven included studies were retrospective trials, and no prospective trials were included. Second, all nine studies were performed in China, and the prognostic significance of ALRI in patients with HCC in other countries remains uncertain. Finally, the cut-off values for ALRI varied across the eleven included studies, and the calculation methods were also inconsistent. Lastly, as the original study did not report underlying liver disease (i.e., the presence of cirrhosis, HBV, HCV, or nonalcoholic steatohepatitis), we could not perform subgroup analysis based on this variable. Hence, an updated meta-analysis including more clinical studies from various areas is still needed in the future.

In conclusion, high ALRI in patients with HCC predicted inferior survival outcomes and was strongly associated with some important features that imply tumor progression in HCC. However, more prospective clinical trials investigating the association between ALRI and survival among HCC patients from diverse ethnicities are necessary to verify this conclusion.

## Figures and Tables

**Table 1:** Major features of eleven references included in this meta-analysis.

Author year	County	Study period	Study design	Sample size	Age (years)	Sex (M/F)	Staging criteria
He (2017)	China	2007-2013	Retrospective	241	50.3	210 (87%)/31 (13%)	Milan criteria
Liu (2020)	China	2011-2016	Retrospective	206	53	160 (78%)/46 (22%)	AJCC 8th
Jin (2015)	China	1997-2008	Retrospective	371	NA	323 (87%)/48 (13%)	NA
Yang (2015)	China	2009-2015	Retrospective	189	53.4	161 (85%)/28 (15%)	BCLC
Zhao (2019)	China	2009-2013	Retrospective	429	54	392 (91%)/37 (9%)	AJCC 7th
Zheng (2019)	China	2011-2013	Retrospective	78	60.12	67 (86%)/11 (14%)	BCLC
Suo (2019)	China	1993-2010	Retrospective	463	50.14	401 (87%)/62 (13%)	BCLC
Qin (2019)	China	2013-2017	Retrospective	191	48.62	154 (81%)/37 (19%)	AJCC 8th
Chen (2020)	China	2007-2016	Retrospective	983	50.5	829 (84%)/154 (16%)	BCLC
Liao (2021)	China	2009-2016	Retrospective	416	50.47	359 (86%)/57 (14%)	NA
Wu (2021)	China	2014-2017	Retrospective	347	NA	290 (83.6%)/57 (16.4%)	BCLC/AJCC8th
Author year	Tumor stage	Treatment	Cut-off value	Cut-off selection	Follow-up months	Survival analysis	NOS score
He (2017)	Size <3; 3-5 cm	Surgical resection	32	Mean value	54.2	OS, PFS	7
Liu (2020)	I-IV	Surgical resection	18.734	ROC analysis	35	OS	8
Jin (2015)	I-IV	Surgical resection	25.2	ROC analysis	20	OS, PFS	7
Yang (2015)	A-C	TACE	57	R software	30.2∗	OS	8
Zhao (2019)	I-IV	Palliative treatments	86.3	ROC analysis	NA	OS	8
Zheng (2019)	B-C	TACE	22.82	ROC analysis	18.16∗	OS, PFS	8
Suo (2019)	0-C	Surgical resection	25.2	ROC analysis	47.12	OS, PFS	8
Qin (2019)	I-III	Surgical resection	26.06	ROC analysis	32.4	OS	8
Chen (2020)	A	Surgical resection	26.6	X-tile	48.8	OS, PFS	8
Liao (2021)	I-III	Surgical resection	22.6	ROC analysis	36.7	O, PFS	8
Wu (2021)	Size≤5; >5 cm	Surgical resection	31	ROC analysis	45	OS, PFS	8

**Table 2:** Subgroup analysis of pooled HRs and 95% CIs between ALRI and OS and PFS in hepatocellular carcinoma.

Variables	No. of studies	No. of patients	Effects model	HR (95% CI)	*P*	Heterogeneity	
						*I* ^2^ (%)	*P*
OS							
Total	11	3914	Random	1.53 (1.25-1.82)	<0.001	80.4	<0.001
Treatment							
Surgical resection	8	3218	Random	1.49 (1.17-1.81)	<0.001	82.7	<0.001
TACE	2	267	Random	2.20 (1.19-3.22)	<0.001	0	0.940
Palliative treatments	1	429	_		0.004	_	_
Staging criteria							
AJCC/NA	6	1960	Random	1.60 (1.38-1.81)	<0.001	0	0.823
BCLC	5	2060	Random	1.56 (1.20-1.91)	<0.001	43.3	0.133
Milan criteria	1	241	_	1.02 (1.01-1.02)	<0.001	_	_
Sample size							
<230	4	664	Random	1.82 (1.29-2.34)	<0.001	0	0.701
≥230	7	3250	Random	1.45 (1.14-1.76)	<0.001	85.2	<0.001
Cut-off value of ALRI							
<26	5	1534	Random	1.51 (1.21-1.80)	<0.001	31.4	0.212
≥26	6	2380	Random	1.54 (1.12-1.96)	<0.001	83.3	<0.001
Cut-off selection							
ROC analysis	8	2501	Random	1.49 (1.31-1.68)	<0.001	0	0.441
R software	1	189	_	2.18 (1.30-3.65)	0.003	_	_
Mean value	1	241	_	1.02 (1.01-1.02)	<0.001	_	_
X-tile	1	983	_	1.76 (1.42-2.17)	<0.001		
NOS score							
<8	2	612	Random	1.24 (0.73-1.76)	<0.001	84	0.012
≥8	9	3302	Random	1.58 (1.37-1.78)	<0.001	16.3	0.298
PFS							
Total	7	2899	Random	1.36 (1.10-1.63)	<0.001	81.8	<0.001
Treatment							
Surgical resection	6	2821	Random	1.35 (1.08-1.61)	<0.001	84	<0.001
TACE	1	78	_	2.21 (1.04-4.71)	0.040	_	_
Staging criteria							
NA	2	787	Random	1.60 (1.31-1.89)	<0.001	0	0.521
BCLC	4	1871	Random	1.37 (1.18-1.55)	<0.001	0	0.651
Milan criteria	1	241	_	1.01 (1.01-1.02)	<0.001	_	_
Sample size							
<230	1	78	_	2.21 (1.04-4.71)	0.040	_	_
≥230	6	2821	Random	1.35 (1.08-1.61)	<0.001	84	<0.001
Cut-off value of ALRI							
<26	4	1328	Random	1.48 (1.21-1.74)	<0.001	18.7	0.297
≥26	3	1571	Random	1.24 (0.92-1.57)	<0.001	85.5	0.001
Cut-off selection							
ROC analysis	5	1675	Random	1.46 (1.26-1.66)	<0.001	0	0.448
Mean value	1	241	_	1.01 (1.01-1.02)	<0.001	_	_
X-tile	1	983	_	1.39 (1.17-1.65)	<0.001	_	_
NOS score							
<8	2	612	Random	1.22 (0.73-1.70)	<0.001	82.9	0.016
≥8	5	1524	Random	1.42 (1.25-1.58)	<0.001	0	0.443

**Table 3:** Correlations of ALRI and clinical factors in patients with hepatocellular carcinoma.

Characteristics	No. of studies	No. of patients	Effects model	OR (95% CI)	*P*	Heterogeneity	
						*I* ^2^ (%)	*P*
Sex, male vs. female	6	1995	Random	1.32 (1.02-1.70)	0.035	0	0.613
Age, years, >60 vs. ≤60	3	475	Random	0.89 (0.56-1.43)	0.637	0	0.630
HBsAg, positive vs. negative	5	1566	Random	1.26 (0.95-1.67)	0.104	0	0.410
AFP, ng/ml, >400 vs. ≤400	4	1418	Random	1.45 (0.89-2.37)	0.136	71.9	0.014
Cirrhosis, yes vs. no	5	1566	Random	1.68 (1.01-2.81)	0.046	63	0.029
Tumor size, ≥5 cm vs. <5 cm	5	1566	Random	2.25 (1.31-3.88)	0.003	81	<0.001
Tumor number, multiple vs. single	4	1360	Random	1.19 (0.87-1.64)	0.271	0	0.685
TNM stage, III-IV vs. I-II	4	1246	Random	1.94 (0.97-3.90)	0.062	87.6	<0.001
PVTT, yes vs. no	4	1069	Random	2.50 (1.52-4.11)	<0.001	47.4	0.127
Distant metastasis, yes vs.no	3	878	Random	1.72 (1.05-2.82)	0.031	0	0.472

## Data Availability

The datasets analyzed during this study are available from the corresponding author on reasonable request.

## Supplementary Materials

Supplementary Materials Table S1 is the detailed items of Newcastle–Ottawa Scale in this meta-analysis.
