# Supplementary material for: Prognostic and Clinical Significance of Aspartate Aminotransferase-to-Lymphocyte Ratio Index in Individuals with Liver Cancer: A Meta-Analysis
Source: Dis Markers. 2022 Feb 9;2022:3533714. doi: 10.1155/2022/3533714 (PMC8850034; doi:10.1155/2022/3533714)
Supplement: Supplementary Materials — Table S1 is the detailed items of Newcastle–Ottawa Scale in this meta-analysis. [file 3533714.f1.docx]

**Table S1 The detailed items of Newcastle–Ottawa Scale in this meta-analysis**

| Research | Selection | | | | Comparability | | Exposure | | | | NOS Score |
| --- | --- | --- | --- | --- | --- | --- | --- | --- | --- | --- | --- |
|  | Is the case definition adequate? | Representativeness of the cases | Selection of Controls | Definition of Controls | Select the most important factor | study controls for any additional factor | secure record (eg surgical records) | structured interview where blind to case/control status | Same method of ascertainment for cases and controls | Non-Response rate |  |
| He^2017^ | 1 | 1 | - | 1 | 1 | 1 | 1 | - | 1 | - | 7 |
| Liu^2020^ | 1 | 1 | - | 1 | 1 | 1 | 1 | - | 1 | 1 | 8 |
| Jin^2015^ | 1 | 1 | - | 1 | 1 | 1 | 1 | - | 1 | - | 7 |
| Yang^2015^ | 1 | 1 | - | 1 | 1 | 1 | 1 | 1 | 1 | - | 8 |
| Zhao^2019^ | 1 | 1 | - | 1 | 1 | 1 | 1 | 1 | 1 | - | 8 |
| Zheng^2019^ | 1 | 1 | - | 1 | 1 | 1 | 1 | - | 1 | 1 | 8 |
| Suo^2019^ | 1 | 1 | - | 1 | 1 | 1 | 1 | - | 1 | 1 | 8 |
| Qin^2019^ | 1 | 1 | - | 1 | 1 | 1 | 1 | 1 | 1 | - | 8 |
| Chen^2020^ | 1 | 1 | - | 1 | 1 | 1 | 1 | 1 | 1 | - | 8 |
| Liao^2021^ | 1 | 1 | - | 1 | 1 | 1 | 1 | 1 | 1 | - | 8 |
| Wu^2021^ | 1 | 1 | - | 1 | 1 | 1 | 1 | 1 | 1 | - | 8 |
